# A Short Treatise on Typhus Fever

**Published:** 1839

**Authors:** 


A SHORT TREATISE ON TYPHUS FEVER. By *George Leith Roupell*, M.D. Physician to St. Bartholomew's Hospital, Consulting Physician to the Seaman's Hospital, &c. &c. Octavo, pp. 301. London, 1839.

FEVER—inflammatory fever—nervous fever—typhus fever—typhoid fever—gastric fever—gastro-enteric fever, &c. &c. &c.—do these and the hundred-and-one terms besides, so frequently met with in medical writings, represent severally a particular and definite state of disease, which can readily be distinguished by fixed and essential characteristics? Alas! no. So vague, on the contrary, are our notions respecting the different forms of fever, and so indefinite and defective our descriptions of them, that after reading two separate treatises on the disease, we are often unable to say, whether the type treated of in each be, or be not, the same. Take, for instance, the works of Louis and Armstrong. Do these authors describe the same species of fever? Yes, answers one learned physician; undoubtedly no, exclaims another, equally learned. What then are we to gather from such discrepancy of opinion? This sad and humiliating truth—that as yet we have no accurate knowledge of the specific characters of fever, much less of those more minute points of difference which constitute its numberless varieties. Nay, truth compels us to go farther still, and add, that as yet we do not possess any undisputed, clear, and definite *generic* description of fever; so that we may well say in the words of Ramazzini, pronounced by him nearly a century and a half since: "Verum quod dolendum est, post annorum chiliades omnes medentium conatus et machinamenta ad febris naturam explorandam, nedum oppugnandam, pene in irritum cesserint. Quoties cum veterum, tum recentiorum medicinæ procerum præstantiora monumenta, et, quæ creduntur cedro magis digna, volumina evolvere mihi volupe est; idem prorsus mihi evenire sentio ac Terentiano seni, qui cum in filii sui causa plures advocatos accersisset, cosque inter se pugnantes deprehendisset, *incertior*, inquit, *multo sum quam dudum.*"

The objects which have been aimed at in the work before us, are thus stated in the preface:—

"To assert the claim of the prevailing epidemic to be ranked among specific fevers, to separate it from some with which it has long been improperly confounded, to show at the same time its analogy with others, and to improve the pathology of all." iv.

After remarking that a knowledge of the seat, character, and progress of a disease, is essential to precision and success of treatment, Dr. Roupell adverts to the vague and indefinite manner in which authors have treated of typhus fever. This disease has been prevalent for several years past in London and its outskirts, and Dr. R. has treated numerous cases, both at the Seaman's Hospital and at St. Bartholomew's. To prevent this, the true typhus, from being confounded, as it too often is, with other febrile diseases, our author proposes "to define accurately the malady to which the term should be restricted, and at the same time to assign to it a proper nosological position." This has not been accomplished either by Sauvages or Cullen; nor has the real character of the disease been elucidated by more recent authors in our own country. The definitions of Cullen, Sauvages, Tweedie, and Copland being quoted, are thus commented on.

"In the above descriptions Typhus is considered to belong to the continued fevers. It is looked upon by the more recent authors in this and other countries not as an individual disorder, but as one into which others may readily be and frequently are converted." 6.

The result of Dr. R.'s observations has led him to a different conclusion. He is convinced that the prevailing fever is owing to a certain specific cause; that it pursues a definite course, passing through its stages with regularity, spreading by infection, and marked in its progress by a distinctive rash. Here, then, we have all the characteristics of the genuine exanthemata of authors, to which class it seems correctly and exclusively to belong. This opinion as to the nature of the disease was stated in a paper read by our author before the College of Physicians in 1831, and was then thought by him to be original. He has since, however, discovered that Professor Hildenbrand of Vienna, had anticipated his views, and to him therefore he resigns the claim of originality.

Without pronouncing, at present, any opinion on the views expressed in the foregoing paragraph, we deem it due to Dr. Copland to remind our readers that, in that part of his article on fever which treats of *typhus*, (Dictionary, p. 1009,) he details fully, and with his usual clearness, precisely the same views and opinions for which Dr. R. is contending. But this latter gentleman, by an extraordinary, and almost unaccountable mistake, has quoted Copland's Definition of *Typhoid Fever*, overlooking altogether that of genuine *typhus*. Either Dr. R.'s own notions respecting the nature and symptoms of the disease about which he is writing, are very loose; or he must have been not a little surprised at the inapplicable description given by the author of the *Dictionary*. The occurrence of such an error is a strong proof of the truth of those remarks with which we introduced our present article, and, if we are not mistaken, we shall find other similar errors as we proceed. But to resume our analysis:—

Professor Hildenbrand's observations refer especially to the epidemic typhus of 1806, but he had studied the disease for twenty years and upwards, under circumstances very favorable to enquiry. He states that simple contagious typhus has eight stages or periods—1st, the *stage of contagion*—this period he deems instantaneous; 2d, that of *incubation*—period from three to seven days; 3d, the stage of *invasion*—duration from six to twelve hours; 4th, *inflammatory* stage—duration seven days; 5th,

*nervous* stage—this continues five or six days, after which ensues, 6th, the *critical* stage, which lasts only a few hours, and is followed by, 7th, the stage of *remission*—during this stage the symptoms gradually abate for a period of seven days, when the patient enters on, 8th, the *convalescent* stage. This runs through several weeks, and has no exact period. The patient, now recovered, is, to a certain extent, exempt from a recurrence of the disease, and occasionally finds that some previously existing ailment has departed with the fever. The symptoms attendant upon the several stages are detailed at large; but we do not think it necessary to transcribe them fully. On the fourth day from the commencement of the inflammatory stage, a purpurous rash appears on the surface of the body, particularly in those parts which are kept warmest. Together with this, the peculiar exanthema of typhus, petechiæ, often appear. The former sometimes disappears in the fifth, or nervous stage; the latter remain and even increase, or now show themselves for the first time if they have not previously appeared. In some cases the rash does not exist at all, or is so little developed as to escape notice. Occasionally it presents itself under the form of miliary petechiæ, and disappears in a few hours; or having been out its usual time, departs without affording corresponding relief. The disease is most infectious in the fifth stage.

There are many circumstances which may complicate typhus, and numerous irregularities may arise in its course; but these are common to typhus with the other exanthemata.

Having quoted Hildenbrand's description, Dr. R. proceeds to consider "whether or not the epidemics which have appeared from time to time, and have recently prevailed, ought to be classed with the disorder thus described." That of 1831, of which an account was published by our author, "presented all the striking features of this malady." Dr. Armstrong, in his description of the typhus of 1817, notices an "anomalous rash," which he had known to be occasionally mistaken for the eruption of measles or scarlet fever. Petechiæ were very common. Huxham, in his account of the epidemic of 1734-5, mentions not only petechiæ but also papulæ and pustules. Sir John Pringle, treating of the fever which broke out during the "black assize" of London, in 1750, mentions *certain spots* which were "frequent but not inseparable attendants of the fever." "These are," he continues, "the true petechiæ," a statement questioned by Dr. R., who concludes, (we think without sufficient reason,) that what Pringle "terms petechiæ, may probably have been the rash usually seen in these cases, or perhaps a mixture of both."

The fever which prevailed at Genoa in 1799 and 1800, presented, according to Rasori, petechiæ, *or an eruption little differing from petechiæ,* or a miliary eruption, or both; from which confused description Dr. R. infers, that the characteristic symptom of typhus must have been present. M. Louis, describing the disease which he terms, "gastro-enteritis, putrid, adynamic, ataxic, typhoid fever," speaks of a rash, rosy, lenticular, mixed with sudamina. The other symptoms of the fever corresponded, says Dr. R. with those described by Hildenbrand.

Were, or were not, the various forms of fever alluded to in the foregoing paragraphs identical? We think not, and we are confirmed in our opinion by the authority of Dr. Copland, who distinguishes the true typhus as well from the *low nervous,* and *putrid* fevers, as from the *typhoid fever* with *prominent gastric affection,* of the French. This distinction, if it be true, (and most physicians in this country will assent to its truth) not only renders useless Dr. R's quotations from Huxham, Pringle, and Louis, but converts them into an argument against his own views, by showing that the rash on which he lays so much stress as characteristic of true typhus, is, according to his own representation of the passages quoted by him, of not unfrequent occurrence in other and different forms of fever. Huxham himself insists strongly on the difference between his *slow nervous fever*—that to which the observations cited by Dr. R. refer—and another type of fever which he terms *putrid malignant.* Yet in his description of this latter, he mentions the frequent occurrence not merely of petechiæ, but of a rash—" *sæpissime occurrit exanthema morbillis simile, &c.*" Here then we have the rash, which we are told to regard as characteristic of typhus, occurring in a fever different from that which Dr. R. assures us was genuine typhus. How is this difficulty to be surmounted? Either the rash is not peculiar to typhus, or both the types of fever referred to were identical. But Huxham, who saw and described them, and whose authority therefore must be conclusive, declares that they were widely different—different in their origin, different in their symptoms, and different in their method of cure.

Nothing, we grant, can be more difficult than to establish satisfactorily either the identity or difference of epidemics, the descriptions of them being in general extremely loose, and the nature of the disease seldom or never defined; for we cannot give the name of definition to a mere detail of all the existing symptoms, whether common or peculiar. But the very difficulty here mentioned ought to make authors more cautious in supporting any favourite views which they may have, by a reference to previous writers, who are perhaps no authority at all on the subject in question. This is but one of the many evils wich have resulted to medical science from the neglect of concise, strict, and apposite definitions. How many totally different diseases have been jumbled together in consequence of assigning too much importance to their *common* symptoms! And how much has not this same circumstance contributed to a slovenly inattention in practice! Very few of the great mass of medical practitioners enquire minutely into the *peculiar* symptoms of diseases, and few therefore ever advance to truly rational methods of treatment. Diseases are recognized solely by their family resemblances; their individual traits are altogether overlooked. How imperfect, and to a rightly-constituted mind unsatisfactory, must such a knowledge of them be!

Has typhus ever been accurately defined? We have seen that Dr. Roupell finds fault with the definitions of Sauvages, Cullen, Tweedie, and Copland. Let us hear what he himself has to say on the subject. Referring to the epidemics to which we have already alluded, he thus proceeds—

"It will not be necessary to dwell longer upon the proof of identity between all the diseases above enumerated. Many of the symptoms are found in all fevers, others again are common to what are called putrid or pestilential fevers, and a third class are peculiar to the disease in question. Each of these orders of symptoms will claim some notice at my hands with the hope of being able to arrive at a general conclusion from particular propositions.

Typhus fever in common with all others, exhibits in the first place all the phenomena incident to symptomatic or secondary fevers. The constitutional febrile symptoms which arise in consequence of local inflammation so closely resemble those of idiopathic fever that they cannot often be distinguished, except by the history of the case or by the local affection.

It occasionally puts on those signs which are supposed to denote putrescence, such for instance as extreme prostration, great tendency to gangrene, fætor of the evacuations, cadaverous smell of the whole body, copious discharges of blood, and a rapid tendency to decomposition after death. Scarlet fever as is well known not unfrequently puts on this appalling character. Putrid symptoms may also come on during the progress of the measles or small-pox. These signs therefore may be called accidental and are common to a variety of specific diseases.

It has in the third place some other symptoms essentially its own; these are rash upon the skin, the power of spreading by infection, and the certain period of duration." 32.

The above division of the symptoms into three groups has an appearance of accuracy, and the whole passage quoted might be considered by a superficial reader as both very clear and satisfactory. And yet it would be scarcely possible to find in any other writer a passage equally calculated to exemplify the illogical vagueness which is unfortunately so common in medical writings. The symptoms of typhus fever "essentially its own, are rash upon the skin, the power of spreading by infection, and the certain period of duration." What! are not these symptoms equally characteristic of measles, of small-pox, of scarlet fever? How then, speaking of typhus, can they be said to be "essentially its own"? Dr. Roupell is known to be a learned man, and when learned men write thus, what can be expected from the common herd?

But let us not proceed too fast. Perhaps the doctor meant, that the abovementioned symptoms, when existing in conjunction with certain other symptoms mentioned, are pathognomic of typhus fever. Well then, let us consider the subject in this light. We have already shown that the symptoms composing the third group are common with typhus, to measles, small-pox, and scarlet fever. Dr. R. himself states that this is the case with respect to the symptoms comprised in the second group; while those of the third, are common to all fevers, and thence of necessity to measles, small-pox, and scarlet fever. Thus *all* the symptoms of typhus fever are common to measles, small-pox, and scarlet fever. How absurd such a conclusion! And yet to no other can our author's statements lead.

Dr. R. devotes five pages to a consideration of the *rash* of typhus, re-quoting the authorities previously mentioned, and in addition also that of Chomel, who states that there is an eruption in the majority of cases. As Dr. R.'s views are influenced chiefly by the frequent appearance of this rash, we think it due to him to transcribe the following statements respecting it.

"There are many circumstances which throw obstacles in the way of obtaining accurate information respecting the rash in typhus. Patients frequently delay their application to hospitals for several weeks, so that in one class of cases it will have disappeared before the disorder came under notice. In another class it will probably be overlooked, for it is often so slight as to escape any but an experienced eye. Then again it will not be perceptible on the chest or arms, where it is usually expected to be found. On one occasion where it had been sought for in vain on the front part of the body, it was perceived abundantly on the back, which was accidentally examined, owing to the necessity of confining the patient in a straight-waistcoat. Little can be learned on this subject, from the patients themselves, when asked if they have any eruption on the skin, they almost invariably say no, and even should it be fully out, are seldom aware of it. Treatment may in some degree interfere with its appearance. Many cases have occurred in which no rash could be detected, after the adoption of active measures at the commencement, especially if emetics were administered on the first symptoms declaring themselves. Independent however of all these circumstances, it appears in the larger number of cases. According to Louis it is seen in two out of three, nay more, in fifteen out of sixteen; Chomel says, it appeared in thirty-two out of fifty-four. By reference to my note-book, I find that the rash is recorded in 70 out of 100 cases promiscuously taken, no mention being made of it in the remaining 30: again, out of 100, all of whom had the rash, it was present in 86 on their admission, and showed itself subsequently in the rest.

The rash then may fairly be considered as one of the characteristics of this fever, and the assertion of Sauvages, that it arises from an over-exciting treatment is sufficiently contradicted by modern experience. For the rash in this as in other eruptive fevers seems to be exhibited more fully, since the general introduction of a cooling regimen has been adopted and sedulously enforced. To Sauvages' objection against placing typhus among the exanthemata, on account of the lateness at which the rash appears, we may oppose the fact, that it is constantly perceived on the third or fourth day.

The eruption itself is of a red colour, the shades of which are various, in some cases bright and vivid, more generally however dusky, and undertoned: if the rash is fully developed the cuticle is slightly elevated, and when the vessels are very turgid the eruption is perceptible to the touch. It is most commonly found on the chest, trunk and limbs; sometimes on the face, and was noticed in a recent case to have reached and occupied the scalp; nor is it confined to the outer surface of the body, but extends itself to the lips and lining membrane of the mouth.

It appears in spots or patches circular in form, and varying in size from the diameter of a pin's head to that of a pea. No itching attends its presence on the skin, nor is desquamation of the cuticle an ordinary consequence. From the appearance of the patients in the eruptive stage, the term spotted is not inappropriate to this fever; and its existence in London in 1750 may be presumed from the fact that the bills of mortality for that year, besides the ordinary eruptive disorders, as measles, small-pox and Scarlatina, announce as prevalent 'Malignant Fever, Spotted Fever, and Purples.' We learn from the same source that in the above-mentioned year the mortality from these causes was very considerable, 1229 persons died from small-pox, 321 from measles, and 4294 from fever. In the following year the number of deaths were proportionately small, 998 from small-pox, 31 from measles, and 3219 from fever. A decrease in the number of deaths from these causes alone of nearly six hundred.

The duration of the rash in Typhus, according to M. Chomel, is from three to four days; should any mottling of the skin appear after this period, he attributes it to a fresh eruption; and remarks that the exanthema disappears occasionally on the second day after it has shewn itself. It may here be observed that in some cases, when the rash could not be perceived, but in which it was deemed advisable to bleed the patient, and a ligature was applied for that purpose to the arm, the eruption appeared below the tape. Acting upon this idea I have at other times been able to exhibit it, by artificial congestion of the vessels." 37.

Our author details eight cases of typhus, intended to illustrate the different periods at which the rash appears. In the first case the disease, if it were really typhus, was mild to a very unusual degree. The patient, already ill two days, was admitted into hospital on the 14th of July, and discharged well on the 1st of August. The rash appeared on the day after admission—the third day of the fever.

The second case was also mild, being unattended with delirium; (is true typhus ever without delirium?) and the rash, ("a copious eruption of dusky red spots") shewed itself on the fourth day.

The third case was complicated with pneumonia—rash on the fourth day.

In the fourth case, "sore throat" was complained of, and the rash appeared on the fifth day.

In the fifth case, "the skin was spotted with a rash" on the sixth day. The late appearance of the rash in this case, Dr. R. attributes to an emetic administered on the first appearance of the symptoms. "In several other instances," says our author, "where the fever commenced in the hospital, and the same treatment was adopted, no rash could be discovered." We shall hereafter have occasion to advert to this circumstance.

The subject of the *sixth* case had been ill six days before admission into hospital. "There was no appearance of rash when he was admitted in the early part of the afternoon, but in the evening, when seen in bed, he was covered with it."

In the *seventh* case, the disease originated in St. Bartholomew's Hospital. An emetic was given, and no eruption appeared.

In the *eighth* case, the rash had not entirely vanished a month after its first appearance. Dr. R. remarks that "the cases are generally severe in which this retardation occurs, and that it seems to be often occasioned by some deep-seated mischief. The rash then, he continues, "may appear at various periods, it may be prolonged some weeks, or it may not appear at all, and I have seen it recede after having been out for a few hours only. We are at no loss for analogous effects in other exanthematous disorders. If M. Rayer's work should be consulted, it will there appear that the eruption in measles will sometimes show itself earlier than usual; for instance on the third day, and even sooner. The same author gives us examples on the other hand of its retardation to the fifth or sixth." In scarlet fever, the eruption, it is well known, is sometimes altogether absent.

Nor can the rash of typhus be confounded with that of any other exanthematous disease.

"It cannot be mistaken for variola, as it never assumes a pustular form; from roseola it is to be distinguished by the following signs, the patch is smaller, it appears later, and there is no itching.

It cannot be confounded with simple erythema, as that is not accompanied by fever; but from measles it is not in its mere aspect to be distinguished, and indeed it may fairly be conjectured that that epidemic, which Sydenham describes as being measles of an anomalous and malignant form, was real typhus. The measles, he says, of 1674 deviated from rule, did not preserve their type, the eruption came out irregularly, was often confined to the neck and shoulders, the bran-like desquamation did not result, peripneumonia more frequently took place, and in some cases the fever would last 14 days and more.

This disease differs from measles in many respects, its duration is longer, it is wanting in the usual precursory affection of the eyes and sneezing; the rash is more irregular in form, it does not pass progressively from the head or trunk to the extremities, and those who have had the measles are not free from an attack of this disorder.

The rash of typhus again differs from scarlatina in being less vivid, the redness is not so diffused, there is more tendency to moisture on the skin, there is usually no sore throat, nor is typhus in my experience ever followed by anasarca." 46.

To the analogies existing between typhus fever and the other exanthemata, already insisted on, Dr. R. adds, on the authority of Chomel, the frequent appearance of sudamina.

Having thus exhibited a full, and, as we believe, a correct statement of our author's views respecting the exanthematous nature of typhus, it may not be inexpedient briefly to enquire how far those views are borne out by the facts which he has adduced in confirmation of them. Do these facts afford a satisfactory and stable foundation for the theory which Dr. R. has built upon them? Unquestionably not. In the first place, it is by no means certain that the disease was the same in the different epidemics to which he alludes. On the contrary, we have both reason and authority for concluding that in some it was widely different.

Secondly, the rash is not accurately described by any of the authors cited, so that it is impossible to say whether it was the same in all. One author (Pringle) does not even mention its existence, and we have only Dr. R.'s inference that it *probably* did exist.

Thirdly, The date of the invasion of the rash varies more than in the true exanthematous diseases.

Fourthly, It is more partial than they, affecting only certain parts of the body.

Fifthly, It does not run a fixed and determinate course—it has no settled period of increase, acmé, and decline; at least we are justified in presuming that it has not, since neither our author, nor any of the writers cited by him, have attempted to describe its progress. Chomel mentions, in general terms, that it continues through a period of three or four days. In one of Dr. R.'s cases, already referred to, it had not disappeared after the lapse of a month. To invalidate the objection of irregularity in the appearance, &c. of the rash, Dr. Roupell refers to the occurrence of similar irregularities in the eruptions of measles, scarlet fever, &c. True, but in these diseases such irregularities are exceptions to what is generally observed: in typhus fever, on the contrary, they are of frequent and ordinary occurrence. There is therefore no analogy between the cases.

Sixthly, The rash is altogether absent in many cases of genuine typhus. It existed in only 70 out of 100 of our author's own cases. Dr. Cowan, in his excellent little work on the "*Vital Statistics of Glasgow*," states that the proportion of patients with eruption varied in each month. Do not these facts prove that the eruption of typhus is merely an accidental complication, (a very frequent one, we admit,) and by no means an essential element of the disease. But not only is the rash alluded to often wanting in genuine typhus; it is often present where typhus does not exist. In the form of gastric fever, which prevails among certain classes of our manufacturing population, an erythematous rash, mixed both with papulæ and pustules, is met with in a large proportion of the cases, and is subject to precisely the same irregularities that we have noticed in the eruption of typhus. This symptom of gastric fever is noticed by both the Franks. "Peticulæ quidem sæpe sæpius de præsentia typhi securum afferunt indicium, item miliaria; *at non ita securum, ut illis solis diagnosis superstrui possit; peticulæ enim non raro febrium gastricarum, vel aliarum sociæ.*"* Thus writes Joseph Frank. John Peter's testimony is still more conclusive. In his description of *continued gastric fever*, we have the following passage:— "Interea tum prius, tum hocce morbi stadio, *frequentissima ad cutem exanthemata, petechialia, miliaria tum alba, tum rubra, cum illis interdum mixta, &c.*† What then? Is gastric fever essentially an exanthematous disease? Dr. Roupell will scarcely go so far as to assert this.

Seventhly, We have our author's own authority for the fact, that the administration of an emetic in the commencement of typhus prevents often the appearance of the rash. Could the eruption of small-pox be thus prevented? Most assuredly not.‡

That we have discussed this question at such length, has been owing to the estimation in which we hold Dr. R.'s character as a physician. But for this, we should have thought it sufficient to allude in general terms to the manifest weakness and inconclusiveness of the arguments with which he has attempted to support his views.

Let us now proceed to the next section of the work, the subject of which is, the contagious nature of typhus, or, to use the words of our author, its "power of spreading by infection."

In this country the majority of practitioners believe that typhus may be propagated by infection; but some have not been able to satisfy themselves upon this point, and on the Continent the prevailing opinion is unfavorable to the idea of infection. Our author alludes to Dr. Gooch's tests of contagion, the two most important of which are the effects of seclusion, and of communication with, or approach to, the sick. The most rigid and complete seclusion does not, it is true, always succeed in shutting out infection. Nor is this totally inexplicable, for it is not difficult to imagine that the subtle agent of infection may be readily conveyed through the air, when we see grosser particles of matter, manifest to our senses, thus transported to

---

* Prax. Med. partis primæ, vol. 1, p. 269. Taurini, 1821.

† De Cur. Hom. Mort. Tom. 1, p. 154. Mediolani, 1813.

‡ We are perfectly aware that Dr. R., in a subsequent page, alludes to a case in which the eruption of small-pox was checked by an emetic. The case, he says, is referred to by Dr. Marsh in the 4th vol. of the Dublin Hosp. Reports. We are not told whether the eruption was altogether *prevented*, or only modified by the emetic. If the latter, the fact proves nothing in Dr. R.'s favor; but if the eruption was altogether prevented, we would then ask how the disease was known to be small-pox.

We may as well allude here to another subject on which our author quotes Dr. Marsh's authority. In the volume of Reports above-mentioned, this latter gentleman relates a case of intermittent fever, in which the disease was caught, he alleges, from a patient labouring under typhus. Now, that a case of intermittent fever occurred in a person exposed to the contagion of typhus, we do not for a moment question; but that such contagion had any thing to do with its production far exceeds our belief. If, however, notwithstanding our incredulity, the intermittent did actually spring from such an origin, it must be a new species, and deserves therefore a distinct proper name. Let us call it then the *Dublin Marsh* Fever.—*Rev.*

enormous distances. During the volcanic irruption (eruption ?) at Sambawa in 1815, ashes were carried by the wind to Java, a distance of 300 miles. "Dr. Prout, in his *Bridgewater Treatise,* gives an account of the remarkable haze which appeared in 1782, which was of a pale blue colour, and occasionally yielded a strong and peculiar odour, during the continuance of which, epidemic diseases prevailed; the same author noticed an alteration in the weight of the atmosphere before the appearance of cholera in 1832, and explains it by the supposition that a gaseous body considerably heavier than the air, occupied its place, and this body he considers as a variety of malaria. Not vegetables only, but minute animals, are said to be thus occasionally suspended. Should this be true, we can understand that by the decomposition of such bodies, disorders may be generated." But our author's object is "not to find a source of disease, but to show how easily and how far infectious matters may be conveyed by this medium. The following incidents may serve as practical illustrations of the above assertion. Sir Gilbert Blane mentions that, an isolated case of scarlet fever occurred amongst a ship's crew at sea, long separated from intercourse with the land. Again the children at the Foundling Hospital have no communication with others, from one year's end to another; yet measles, and small-pox appear from time to time among them." Dr. Heberden relates that a man who had been several months in the Penitentiary at Millbank, having no communication, by letter, or by clothing, with any one except the persons belonging to the establishment, who were all free from small-pox, was attacked by that complaint, which, however, at the time was prevalent in the neighbourhood.

The above facts, particularly the first, render it probable that infectious diseases may, and do propagate themselves widely, through the medium of the atmosphere; but it must not be overlooked that they admit also of other explanations; and with regard to typhus fever, we have very strong evidence to prove that the range of contagion is very limited.

"Our chief reasoning then must be founded on the facts which we observe when persons in health approach those who are infected, or when disease appears in a healthy situation, immediately upon the arrival of an infected person; hence in the first place we should look for the propagation of a disease to those in attendance on the sick, and should expect to find that nurses and medical practitioners would be the greatest sufferers." 51.

To shew that such has been the case, Dr. R. alludes to the death, in one year, of Mr. Cook, Dr. Fergus, Dr. John Home, Dr. Johnson, and Dr. Sims, while many other practitioners were attacked, but survived.

"At St. Bartholomew's Hospital alone six pupils were attacked during the last session, and about as many during the one immediately preceding. Among the nurses in attendance upon the sick in that establishment infection was almost universal. Two of the superintendants in one of the medical wards in quick succession were attacked and died. Others were seized, but recovered. Of the whole number of the inferior nurses who were taken ill I am not exactly certain, but seven were under my own charge." 52.

All these facts, which correspond strictly with the statements of Dr. Tweedie respecting infection in the London Fever Hospital, and, we may add, with the records also of that excellent institution the Fever Hospital of

Glasgow, warrant us in believing that typhus fever spreads by infection, being propagated from person to person.

But a still more conclusive proof remains, namely, the propagation of the disease in a healthy situation by the arrival of infected persons. During the Winter of 1830 and the Spring of 1831, great distress prevailed amongst the lower classes of maritime men, to alleviate which, a Refuge was established on the North side of the Thames. It was soon crowded to excess, and, fever becoming prevalent, the inmates were removed by boat-loads at a time to the Floating Hospital. Before their arrival, cases of fever existed on board, yet they were of an ordinary character, and did not present the peculiarities exhibited by those received from the Refuge. But—

"The disorder thus imported soon spread itself over the ship, patients admitted for surgical and other complaints were attacked, the residents on board suffered from fever of a similar kind, and the immediate attendants were severely visited. There were seven nurses employed in the medical wards, whose duty it was to attend on the fever patients, and who returned home when off duty to their families on the south bank of the river. Six of these nurses were attacked with fever of this new type, which a third time made its appearance in a fresh situation, viz. in their houses on shore. The symptoms were the same in each of these different places, and the disease in all was to be distinguished by the characteristic rash. Other circumstances too, both positive and negative, may be mentioned, which confirm my belief that this disease spread by infection. All the attendants at the Refuge, both medical and others, were more or less severely affected by it. Again there was no such disorder in vessels moored near the floating hospital. Nor was there any such fever on the South bank of the river before it appeared in the hospital ship, as I was informed by Dr. Sutton, the physician to the Kent Dispensary, and by other practitioners in the neighbourhood." 54.

Some facts mentioned by Morgagni would lead us to infer that the dead body of a person who had died of fever, is capable of propagating the disease; but Dr. R.'s observations in St. Bartholomew's Hospital tend to show, "that typhus, readily communicable during life, ceases to be so to any serious degree afterwards."

Having discussed the mode in which typhus is propagated, our author proceeds to consider the "causes of its production."

The notion is generally entertained that under certain conditions the poison of typhus can always be generated. Thomson, Hildenbrand, Blane, Pringle, &c. state it as their opinion, that air overcharged with human exhalations is the cause of typhus, hospital, or jail fever. But the facts adduced in proof of these statements do not appear to our author quite satisfactory.

"There was no such disorder amongst the prisoners at Oxford when the black assize was held there in 1577, on which occasion judges, gentry, and almost all who were present perished to the number of 300; the persons in the jail alone we are told were not injured by it. Passing over the improbability of prisoners giving rise to a disease under which they did not themselves labour, it appears that a malignant fever was at the time prevalent in Oxford, to which we are told that '200 more persons of note fell victims besides numbers of lower degree.' Again we are informed that in 1750, when the two judges, the Lord Mayor, one of the aldermen, one sheriff, two or three of the counsel, several of the jury, and above 40 more died, no uncommon sickness was observed amongst the prisoners who came to the bar or were in Newgate. Now it seems clear that a very severe fever, if not this very disorder, was then prevalent in London." 61.

That vitiated air, bad food, and all debilitating causes will predispose the system to receive infection—that the influence of such causes will lead to the extension of any disease propagated by infection, our author admits; but at the same time he denies that such causes are alone capable of producing an infectious malady. Several striking instances, in which extreme filth and closeness failed to originate typhus, are noticed, after which he thus sums up his arguments.

"All then that we can strictly infer is, that debility will predispose to the disease, but as it exists in those previously in robust health, and as when debilitating causes are present it is not always occasioned, we must look for some active, special, and specific virus. The germ of this disorder will not, I believe, be often wanting, for in some of its various forms it seems to be almost always in activity in some portion or other of the world, and amongst all civilized communities of whose disorders we have any accurate account. And when we consider how readily the poison may be conveyed either by the atmosphere, or goods, and the minute particles which may excite the disorder in those susceptible of its influence, we may more properly consider the disease as extended by a virus in actual operation, than suppose it to be recently generated on every fresh outbreak." 64.

Dr. Roupell alludes to the opinions of M. Broussais and M. Louis, on the cause and origin of typhus. It would be idle to detail these here, as they must already be familiar to the readers of this Journal. Besides we have shown that the typhoid fever described by French writers, is a very different disease from the typhus which prevails in these kingdoms. In this latter, lesions of the digestive mucous membrane are comparatively rare; and when they do exist, they would appear, from the train of symptoms, to be for the most part only secondary and contingent.

It would be a strong point in favour of the specific nature of typhus, if it could be shown, that after one attack the system was no longer susceptible of the malady. Hildenbrand considers that for a certain time the disease is not liable to recur, but that this protection does not last during life. An instance is mentioned by him in which typhus proved fatal in the last of three attacks, which followed one another in rapid succession. A distinguished member of our profession has suffered on seven distinct occasions, and another has had three attacks.

"These occurrences are not however conclusive against placing typhus amongst the Exanthemata. For exceptions to the protective power of a first attack are common both in measles, scarlet fever, and small-pox, and examples will probably multiply as our knowledge becomes more perfect and our diagnosis more precise." 67.

Children and aged persons would appear to be less frequently the subjects of typhus. Some authors have claimed for them an extire exemption, but without sufficient grounds for such an opinion. Our author relates three cases of the disease, of the subjects of which, two were children three years old, and one a man aged 53 years.

"*A certain Period of Duration.*"—Under this head our author presents us with some observations, the general tendency of which we cannot certainly comprehend; for, in them, he appears to us not only to appeal to contradictory facts, but to express also contradictory inferences. He begins by stating that "another argument in support of the specific nature of typhus may be derived from the length (*definite* length, we suppose) of time occupied by the disease in its progress;" and then quotes certain authors to show that the usual period of termination is about the twenty-first day. But M. Louis, whose authority he cites, states that the disease occupied a space of from eight to forty days. Hildenbrand assigns to it a duration of fourteen days; and our author himself relates three cases, in two of which the symptoms were at an end within one week, while in the third, convalescence did not begin to take place until the nineteenth day. "These cases," adds Dr. R. "are quoted with a view of showing the termination of typhus at different periods;" and yet, as we have already noticed, he sets out by deriving an argument in support of the specific nature of typhus from its *certain period of duration.* We cannot comprehend our author's object in thus at once arguing *for* and *against* the same proposition. It is by no means difficult to understand what inference he *wishes* to draw, but the adverse facts, it would appear, were too prominent to be left altogether unnoticed.

Dr. R. expresses his belief that the milder cases, above alluded to, as having terminated within a week, exhibited the features essential to true typhus; and in attempting to show the contradictory nature of his statements, we have, of course, been obliged to adhere to his own views. But we are by no means convinced, from his very brief statement of symptoms, that his opinion as to the nature of the disease is correct. To prevent, however, all possibility of misrepresentation, let us give in our author's own words the details of two cases, calculated, we think, to prove the reasonableness of our doubts. Indeed, Dr. R. himself, speaking of the class of cases to which those about to be cited belong, says, "that they could not be positively pronounced to be the disorder now under consideration," and yet he subsequently draws inferences from them, as if the disease was undoubtedly typhus.

*Case* XV.—"George Main, aged 25, had been into one of the Asylums for the Destitute, where fever was prevalent, and was admitted into the Seaman's Hospital on the 2nd of April, 1831. He had frequent rigors, with pain in the head, back, and limbs, his sleep at night was disturbed, his skin was somewhat warmer than natural, but his pulse was quiet, and there was morbid appearance on the tongue. He had been ill two days. The fever quickly subsided, and on the 6th he was pronounced convalescent.

*Case* XVI.—"John Clarke, on admission into the Seaman's Hospital, March 4th, 1831, stated that he had been for some time in the Asylum for the Destitute. He complained of frequent chills, with pain in the limbs, and cough. His tongue was clean, pulse 70, weak, skin cool, bowels loose. The head-ache continued for a few days with confusion of intellect and slowness in answering questions; after which all completely subsided in a short time. He was declared convalescent on the 8th of March."

"These," says Dr. R., "are some of a vast number of instances which, from the general outline of the symptoms, and from the circumstances under which they occurred, justify the inference that they arose from some specific cause. The disturbance of the head, the character of the pulse, the state of the tongue, and the condition of the skin, were not such as would be produced by the accession of simple fever; but such as might arise from the modified virus of some specific disorder, such as typhus." 77.

We cannot, however willing, assent to the truth of these remarks. We have repeatedly known symptoms, similar to those described, exist under circumstances in which the idea of infection could not be entertained. In fact, simple gastric derangement will give rise to a completely corresponding train of symptoms, in ill-fed impoverished patients. Scientific accuracy requires, that in classing diseases, we have some more certain foundation to build upon than a supposed community of origin. We insist on this point the rather that medical men are too prone, during the prevalence of any epidemic, to confound with it every existing disease that bears the least general resemblance to it. How many cases of pleurisy and pneumonia have we known to be treated as influenza, during the prevalence of that epidemic! It will be vain to expect any great improvement in the general practice of medicine, until physicians shall have learned to regard diseases as they really exist, and to reason upon their obvious and sensible characteristics, instead of fashioning for them, as they now but too often do, an origin and a nature, derived in a great measure, if not entirely, from their own imagination.

Among the more serious complication of typhus, our author notices hæmorrhage, erysipelas, suppuration and gangrene.

"Hæmorrhage may take place *either* beneath the skin, from mucous or serous surfaces, *and* (or) even in muscular structure. When beneath the skin, it assumes the form of vibices or petechiæ; which sometimes present themselves in conjunction with the rash, at other times appear as it were instead of it." 78.

Four cases are given in illustration of these statements, after which Dr. R. proceeds to remark, that while true petechiæ are of very common occurrence in typhus, the characteristic rash of the disease is often mentioned by authors under that name. He alludes also to the *conversion of petechiæ into the rash*, or rather to the very contrary, if we mistake not the *intended* meaning of the passage. But let him speak for himself.

"Their conversion into the rash is spoken of by some authors as a common occurrence, and we might *à priori* consider it as likely to be the case, for we should certainly expect that effusions of blood would more readily take place from injected than from other parts." 81.

There can be no doubt that the error lies in the first part of the sentence, and that our author meant to speak of the *rash as terminating in petechiæ*.

Another form of hæmorrhage frequently met with, is epistaxis. This, like hæmoptysis, occurs for the most part early in the disease, while hæmorrhage from the bowels comes on at a later period. Bronchitis is constantly met with in typhus, and the morbid secretion often contains streaks of blood. In the fever of 1831, Dr. R. remarked that the hæmorrhage from leech-bites was very profuse. Hæmorrhage has been ascribed to an altered condition of the blood, and this is no doubt one of its causes. Its frequency in the

*morbus cæruleus* proves, indeed, how much an altered condition of the blood may favor its occurrence; but, at the same time, Dr. R. believes that the state of the vessels themselves in typhus, and the other eruptive fevers, deserves more attention than it has hitherto received. He further adds, that in typhus, "hæmorrhage takes place at one of two distinct periods, the first on or about the third day from the attack, the second upon the fourteenth day, or later." Some cases are given illustrative of the occurrence in typhus of various forms of hæmorrhage. We transcribe the following, in which the morbid appearances were highly interesting.

*Case.*—"John Michael, aged 16, was brought to the Seamen's Hospital on the 14th of January, 1832, in a state bordering upon insensibility; he was constantly throwing about his limbs, his face was pale, the pupil of the eye was dilated, his breathing was oppressed, he had cough with expectoration of blood, and shrank as if he felt pain when pressure was made on the abdomen; the surface of the body was covered with petechiæ. All that could be ascertained about him was that he had had fever, with great pain in the head, and that he had been delirious for a week.

He died very soon after his admission into the hospital. It was observed that the body retained its warmth 24 hours after death. It was covered with purple spots, which were almost entirely confined to the anterior portion of the body.

The liver was greatly enlarged and pale. The spleen was soft and larger than natural. The lungs were healthy in structure, and with the liver and spleen were dotted with petechiæ.

Blood had been effused into the left side of the brain at the lower and back part; the upper portion of the spinal chord was surrounded with blood; there was effusion of blood also into the substance of the left pectoral muscle.

The inguinal glands were enlarged and red, and when cut into yielded blood mixed with a purulent fluid." 87.

*Erysipelas.*—"During the prevalence of the epidemic in various years, idiopathic erysipelas has been a very common and a very serious addition to other complaints in persons exposed from their situation in hospitals and elsewhere to the vicinity and infection of typhus, and so common was it in the progress of the fever during the present year, as well as during that of 1831, that no doubt could be entertained that it was essentially connected with and incident to this disorder. Dr. Bateman noticed it in the House of Recovery, and considers it an accessory disease. M. Louis observes, that shivering rarely took place in the course of the disease except to usher in some new calamity, such as erysipelas. It seems to arise at two periods, of which one is within the first week, usurping as it were the place of the ordinary rash. It prevails at the same time as typhus, is preceded by the same symptoms, and arises amongst nurses or those in attendance upon the patients ill with that fever." 88.

Erysipelas frequently seems beneficial in the later stages of typhus, for, on its invasion, a train of anomalous symptoms will often at once disappear. Cases of this kind are given, and also others in which erysipelas alone presented itself, in persons who had attended patients labouring under typhus, some of whom had erysipelas, and some had not.

The inflammation in typhus frequently terminates in suppuration. Collections of pus form in various parts, especially about the face, neck, and head, and still more frequently in the ear. Several cases illustrating this complication are given, but we have not space to transcribe them.

The following Case, however, deserves to be noted, inasmuch as it shows that, in typhus, hopes of recovery may be entertained under circumstances apparently the most desperate.

*Case* XXXV.—"Anne Clanny, aged 28, having been ill three weeks, was admitted into St. Bartholomew's Hospital, on the 9th of December, 1837, with a flushed and dusky countenance, pains in the limbs, violent pain in the head, with throbbing of the temples, uneasiness in the abdomen, increased on pressure, urgent diarrhœa, a dry, brown tongue, and teeth covered with sordes. The spotted rash was present, was marked and distinct. The chief peculiarity in this case was the urgency of the symptoms referable to the abdomen and the character of the evacuations. The abdomen was much distended, extremely painful, there was constant diarrhœa, and the stomach was so irritable that neither medicine nor nourishment could be retained. The discharges from the bowels, which were copious, consisted of yellow purulent matter of a fœtid odour. Her recovery was extremely problematical for more than a week. She was extremely feeble, yet could not be kept in bed without force, and then talked wildly. Her countenance became livid, her extremities cold, her skin clammy, her pulse mounted up to 140. The fur on her tongue became black, the muscles of the face were convulsed, her eye looked glassy, the evacuations from the bowels still appeared purulent, and with the urine were passed unconsciously. A change for the better took place on the 17th of December. She slowly recovered, and left the hospital on the 26th of January." 96.

Our author makes some remarks on the *nature* of the inflammation in typhus, and quotes from Heberden, Hunter, Thomson, &c. passages which, to us, do not appear to bear very closely on the point in question. He next relates cases in which carbuncle, extensive sloughing, &c. occurred during the progress of the fever. In one case, all the soft parts of both lower extremities mortified, and both legs being amputated, the patient recovered.

The serous membranes are often implicated in typhus.

"Although the inflammation excited in typhus most frequently involves the mucous membranes and cellular tissue, and has a great tendency to suppuration or gangrene, yet this is by no means invariably the case. The serous membranes are often implicated, and the ordinary products of increased action in those parts are not unfrequently produced, forming complications of peritonitis, pleuritis and meningitis. A great many examples might be given, but it will be sufficient to cite a few only to illustrate this statement." 105.

Of these we copy the following, which is given as an instance of peritonitis complicating typhus.

*Case* XLII.—"Martha Dean, aged 20, was admitted into St. Bartholomew's Hospital on the 4th of January, 1838.

She had been ill a week. Her illness commenced with pain in the limbs, epigastrium and chest; purging of dark-coloured matter, with thirst, loss of appetite and great heat of skin. Menstruation had been suppressed for three months, she was not enceinte. Her symptoms on admission were pain in the chest and epigastrium, coldness of the extremities, swelling of the right leg, tongue coated in the centre with a brown fur, bowels relaxed, evacuations dark, no pain in the head, no unnatural sound to be detected by auscultation in the chest, pulse 128 and very compressible. There was in this case a mixture of the rash with petechiæ. On the 5th, menstruation appeared. On the 6th she was reported to have slept well, there had been no evacuation from the bowels, the pain in the epigastrium was less. She continued to improve until the 13th, when she was attacked by shivering followed by pain in the abdomen, the pain was increased by pressure and deprived her of all rest, her pulse 130 and very feeble, her tongue dry and brown, breathing was performed by the respiratory muscles alone. No relief was obtained, and she sank on the 14th.

On examination post mortem the abdomen was found to contain a considerable quantity of turbid fluid mixed with flakes of lymph, and the intestines were coated with a layer of recent lymph which glued them slightly together.

There were several patches of ulceration in the ascending portion of the large intestines.

The other viscera were sound with the exception of the fallopian tubes which were observed to be filled with pus.

No perforation of any portion of the intestinal canal had taken place.

There were no morbid appearances either in the chest or head." 107.

Now, we would ask any unbiassed man of judgment, whether, in the case just related, the abdominal lesions of themselves were not fully sufficient to account for all the symptoms, without supposing the existence of specific typhus? It will be found, on examination, that from the very commencement, and throughout the whole progress of the case, the leading symptoms were referable to the stomach and bowels; while pain of head, delirium and stupor, were absent, or at least are not mentioned. We are told, indeed, that there was "no pain in the head." There were, it is true, the rash and petechiæ, but few physicians, we think, will join Dr. Roupell in attributing so much importance to their presence. The gastro-intestinal derangement will account satisfactorily for the former; while petechiæ, it is well known, may present themselves in all diseases which weaken the *crasis* of the vital fluid.

It is with regret that we thus call in question the correctness of Dr. R's. discrimination, or, we should rather say, classification; for on points of practice, we doubt not that he distinguishes with sufficient accuracy. But when men *write* about diseases, they do not always display the same judgment and caution which they employ in *treating* them. Either they are blinded by some favorite theory, against the evidence both of reason and observation; or failing through negligence to consider well the subjects which they discuss, they throw their remarks together loosely to the great waste of their readers' time and patience, and the incalculable detriment of science.

Of pneumonia occurring as a complication of typhus, our author relates two cases out of many, and then proceeds to consider the state of the pulse in this disease. Rasori says that it varies extremely, and Dr. R. gives a few cases confirmatory of the truth of this statement.

We have thus completed a faithful and tolerably full analysis of that part of the work, (extending over 116 pages,) which is devoted more especially to a description of the symptoms of typhus. Of the remaining pages, however, between 50 and 60 are occupied with a re-consideration of these symptoms; and present, together with a repetition of many of the facts previously stated, certain new observations and reflections, to some of which we purpose briefly to advert.

Typhus, our author remarks, may terminate at the end of a week, with mild yet characteristic symptoms; or, in a somewhat severer form, it may be prolonged through the second week, with but little indication of any serious organic change.

"Should, however, the disease be aggravated to a third degree, a more formidable train of symptoms present themselves, such as blackness and dryness of the tongue, subsultus tendinum, tremors of the limbs, local inflammation, erysipelas, gangrene, sloughing, low muttering delirium, which are too frequently the forerunners of death. If then typhus can and often does exist without exhibiting these last mentioned exaggerations of its features, we are justified in considering them as something superadded, or as an aggravation of a disease which may be confined to a much more limited course. And here we may obtain important instruction as to the cause and treatment of these symptoms from collateral knowledge about the same phenomena, already familiar to us from their presence in other diseases." 117.

To what other diseases does Dr. R. chiefly allude? Let himself speak.

"After venesection, parturition, or injury of any part of the body, there follows more or less constitutional excitement, with a tendency to the formation of pus, accompanied by a dry and hot skin, black tongue, muttering delirium, subsultus tendinum, stupor, and many of those formidable symptoms that characterise severe attacks of typhus. These symptoms are now properly referred to inflammation of the inner lining of the veins, and it seems therefore a fair inference to be drawn, that although the cause in the two cases may be different, yet that the effect in both is the same; and that both in typhus and the peculiar form of typhoid fever which results from injuries, inflammation of the vessels, and the consequent admixture of morbid secretions from the inner membrane with the blood, is the cause of the serious and alarming symptoms which arise, as well as of the secondary lesions." 119.

And again—

"Thus we see that inflammation of a vein, and admixture of morbid secretions from the inflamed part with the blood, causes, on ordinary occasions, typhoid symptoms, and gives rise to a fever attended by subsultus tendinum, low muttering delirium, and a black tongue. The similarity of the symptoms in phlebitis, and in one stage or period of typhus, gives reasonable ground for belief that the corresponding appearances in the two diseases arise from a not dissimilar cause, and hence we see an explanation of one great train of effects in typhus, and learn how typhoid symptoms appearing in various diseases, both chronic and acute, may easily and naturally be confounded with real typhus. Hence also we see how readily what is called spontaneous typhus can be generated. This disease is said always to accompany an army, but phlebitis, exhibiting urgent constitutional symptoms, may probably have been mistaken for it." 125.

We were somewhat startled by the unqualified statement contained in the first sentence of the former of these paragraphs, which, if only its expressed meaning be regarded, must lead the reader to imagine that the grave symptoms there detailed, are the *usual* consequences of venesection, parturition, &c. But passing over this, let us consider whether the inference drawn by our author, be, as he represents it, a fair one. On what data does he ground this inference? Has pus been found in the veins of patients who have died of typhus fever? Have "morbid secretions from the inner membrane" been met with, either attached to it, or mixed with the blood? Again and again must these inflammatory results have been observed, if they really exist in typhus. But alas! for our author's views, no such arguments as these can be adduced in their favour. What then? Are those views altogether unsupported? No; that would be too negligent a proceeding even for a downright theorist. It is urged upon us that the symptoms which characterise severe attacks of typhus, are the same as those attendant on phlebitis. Well, grant that they be: does it follow that "although the cause in the two cases may be different, yet the effect (namely, admixture of pus, &c. with the blood) in both is the same?" If this were acknowledged with regard to phlebitis, we might pursue a similar line of argument with respect to that form of pneumonia which occurs in unhealthy exhausted patients, low gastric fever, pestilential intermittent fever, &c. &c. In fact, there is no asthenic disease whatever, which may not, towards its close, present the train of symptoms generally denominated typhoid. Will Dr. R. contend that in all such cases phlebitis exists? If so, we should like to have his proofs, failing which, we must continue to believe that the graver symptoms of typhus are no more produced by phlebitis, than they are by asthenic pneumonia, low gastric fever, &c. &c.

After these remarks we cannot but look upon Dr. R.'s long quotations on the subject of phlebitis as misplaced, and therefore altogether useless.

*Hæmorrhage.*—The frequent occurrence of this symptom in typhus has already been noticed. It may present itself at an early or a late period of the disease. In the former case, Dr. R. thinks that it depends in a great measure on increased activity of the vessels; in the latter, on changes in the circulating fluid itself. These two distinct forms of hæmorrhage, occurring as they do under widely different circumstances, cannot, Dr. R. affirms, be too carefully distinguished from each other. This remark is equally applicable to the hæmorrhage of purpura, which presents itself in two different forms, very distinctly marked, and of opposite characters. Several cases of the disease in the *sthenic* form were, some years ago, treated in St. Bartholomew's Hospital by venesection, and with the best results. A case related by Dr. Latham, in the first volume of the Medical Gazette, illustrates the good effects of depletion in the form of purpura here alluded to.

*Erysipelas.*—"The next subject which claims our attention is the connexion of erysipelas with typhus. This occurs very frequently, so frequently indeed that it cannot be regarded as a fortuitous event. It appeared in one-seventh of the cases under my care in 1831, and quite as often in the later epidemics, nor was the proportion less among the cases recorded by M. Chomel. It is remarked by Dr. Tweedie, that in the 'London Fever Hospital, as well as in general hospitals, erysipelas is by no means uncommon. Of protracted cases of malignant fever especially, it is a frequent and a dangerous consequence.' In one case that has been quoted, a man suffering from erysipelas communicated it to a fellow-patient who was attending upon him ; nor are other examples wanting to me where both medical men and hospital nurses have been attacked after similar exposure. Sometimes, indeed, when produced by the typhoid poison, erysipelas appears as the primary affection, in which case its origin is not unlikely to be overlooked, and the disease to be considered as an idiopathic form of erysipelas. It most commonly, however, shews itself in the third week of typhus, and often earlier even when signs of amendment have declared themselves. If it does not shew itself spontaneously, the slightest cause induces its appearance. Commencing usually in the form of a red band across the nose, it quickly extends itself over the rest of the face, then upwards to the head and scalp ; the eyes are closed ; vesication takes place ; and matter is frequently formed in the subcutaneous cellular tissue. When thus produced, it seldom extends beyond the face and head, and varies in its attack from mildness to extreme severity. Whe[illegible]

it takes its rise from parts previously or recently irritated, as by cupping or other causes, or from excoriations, especially those about the nates, it extends over a wider space, involving sometimes the whole trunk; but in whatever situation it may appear, or however excited, it almost always becomes phlegmonous, terminating in the formation of pus. The appearance of erysipelas cannot but be considered salutary in some cases, as headaches will often disappear and convalescence proceeds rapidly afterwards, in others again it is the immediate cause of death. No doubt can, I think, be entertained that this form of erysipelas is contagious: it seems, indeed, to be one of the shapes that typhus can assume, and the conjecture may be allowed that this is the epidemic form of the disease; for it is notorious that those seasons which give rise to typhus generate also erysipelas.

I have cited a case to show that typhus can produce erysipelas, but as yet have been unable to satisfy myself of the possibility of one person contracting typhus from another labouring simply under erysipelas." 134.

Such a case, Dr. R. informs us in a note, has been mentioned by Mr. Ingleby, in a paper contained in the Edinb. Med. and Surg. Journal for April 1838. As we do not happen to have the number at hand, we are unable to ascertain what grounds Mr. Ingleby had for believing that the contagion of erysipelas had produced typhus. We should be inclined to weigh his arguments well before assenting to his doctrine. Our author offers some observations intended to shew that erysipelas is produced by inflammation of the veins, and quotes several authorities in confirmation of this doctrine. But of all the authors cited only one, namely M. Ribes, says any thing which bears directly on the subject. We cannot understand what M. Rayer's remarks on the malignant pustule, or *anthracion*, have to do with that form of erysipelas usually met with in typhus. Indeed M. Rayer himself, as Dr. R. afterwards informs us, having examined several cases of erysipelas, failed to discover any traces of inflammation in the neighbouring veins.

The frequent occurrence both of hæmorrhage and erysipelas in typhus, and the true exanthematous diseases, has already been mentioned by our author, and is again strongly urged by him as an argument in favor of the views which he advocates.

We pass over Dr. R.'s farther remarks on "suppuration," "gangrene," "the peculiar inflammation of typhus," and also "the state of the pulse," inasmuch as they contain little worthy of notice, in addition to what has been said in previous parts of the work.

*Putrid Symptoms.*—A proneness to become putrid appears natural in many constitutions, in others it may be acquired by particular circumstances, but typhus and the other exanthemata seem invariably to develop or generate the condition in all. The putrid diathesis may be engendered by atmospherical causes, and still more so by certain habits of life.

"Passing at once to the condition of that class in which a disposition to putridity is most especially shown in disease, we meet with it amongst those compelled to live in dark, ill-ventilated, and crowded apartments. We find it in persons whose limited means and hard-earned wages deny them wherewithal to allay the bare cravings of hunger, still less to guard against the inclemencies of the weather, and the vicissitudes of an ever-varying climate, and on whom, lowered by privation and exposure, despondency exerts her baneful influence. Now what effect does want of light and fresh air produce on sanguification? What causes the pale cheek and haggard look? The answer to these questions is given us by M. Andral, who observes in his pathological anatomy, that there are certain morbid conditions in which before life has ceased the laws which regulate all matter overcome the resistance of vitality, and while consciousness remains and life still lingers, the system loses its power of generating heat, chemical affinities begin to exert themselves, and putrid symptoms result, these he refers to depression of nervous energy, and then goes on to notice the different modifications of external influences, which more or less are in constant operation upon our frames, such as exclusion of the sun's rays, living constantly in a damp situation, and imperfect nutrition of the body: occupancy of unhealthy places, or deficient alimentation, at once strikes at the functions of the lungs and skin, the direct and indirect organs of sanguification; wasting ensues, the circulating fluids are impoverished, the blood becomes thin, watery, deficient in fibrine, and palpably disordered." 151.

Dr. R. strenuously combats the theory which regards typhus as the result of mere debility, and asks how the advocates of this theory can account for the inflammatory results so frequently witnessed in the brain, lungs, intestines, &c.

"A theory," he remarks, "which ascribes the origin of typhus to the operation of a specific poison producing a series of morbid actions, and provoking inflammation of a modified character, must at least be less injurious in practice than that doctrine which ascribes all the processes of this fever to so insufficient a cause as debility." 155.

But putrid symptoms, though they always appear in the protracted and severer forms of typhus, are yet not absolutely essential to that disease. They must, however, originate in some cause, and Dr. R. again attributes them to vascular inflammation.

"Should inflammation of the minuter vessels be admitted as an early step in typhus, an explanation will at once be afforded us of the various consequences which ensue." 158.

Is it come to this? "Should inflammation of the minuter vessels be *admitted*"—we thought our author had long ago *proved* its existence, at least to his own satisfaction. In the following passage he again expresses himself with confidence as to the truth of his theory.

"It is one of my objects to show that typhus is an eruptive disease. The simple appearance of a rash proves an alteration in the natural condition of the vessels of the skin; that this condition is inflammatory, the symptoms which accompany it sufficiently attest." 159.

This passage, literally interpreted, infers that "the natural condition of the vessels of the skin is inflammatory." Of course Dr. Roupell does not mean this:—he ought to have inserted the word "*altered*" between "this" and "condition."

But notwithstanding all that has been said about the veins, Dr. R. does not consider it a matter of much moment to enquire whether their minute ramifications, or those of the arteries, are the seat of the inflammation. He nevertheless enters upon the enquiry, which he pursues in a manner worthy the reader's notice, for it is a rare specimen of that very convenient mode of reasoning which is commonly called "blinking the question." A great deal is said about inflammation of the veins, and inflammation of the arteries; and authors are quoted to shew that the former is of more frequent occurrence than the latter, and that its tendency to spread is greater; and a case is cited from M. Gendrin, in which pus was formed "in the *arteries* of a man who had died from *fever in consequence of a wound in the hand;* and another from Portal, in which the *aorta* of a young man who had *died from measles* was found to be inflamed:" but with regard to the pathology of typhus fever we have only one solitary observation. It is the case of a patient examined by Dr. R., in whom "there were undoubted signs of inflammation *in all the larger arteries,*" the *vasa vasorum* being seen distinctly to ramify "upon the aorta and its branches to a second and third degree." Such are the arguments by which our author labours to prove, that *phlebitis* is the cause of all the more grave symptoms of typhus!!

*Nervous Symptoms.*—Dr. R. proposes to investigate "a few of the peculiarities in typhus, which appear to originate with the head," not to discuss all the cerebral alterations which occur in that disease.

"The affection of the intellect in patients labouring under typhus is singularly curious and almost peculiar to that fever: they get into a state of the highest nervous excitement, are full of apprehension and suspicious of those around them, charge the attendants with intention of destroying them, and refuse to take medicine from a fear of poison: they exhibit apprehension of imaginary danger or anxiety for self-destruction, and unless carefully watched will throw themselves headlong out of window, or find other means of accomplishing suicide. There is in most the greatest despondency, in others, however, though very rarely an unusual degree of hilarity. In this fever, what are called the higher faculties of the mind are less affected than the lower: judgment and power of connected reasoning constantly remain when the memory has entirely gone. Hildenbrand states, that being in this condition he conversed about himself with his medical attendants, and made sensible suggestions upon the treatment of his own case; there is indeed a connexion in the answers of typhus patients with the questions put to them, which is often extraordinary, though they afterwards have not the slightest recollection of what has passed, nor sometimes whom they have seen, though they recognized acquaintances at the time. The nervous system exhibits peculiarities as well during convalescence as in the earlier periods of the disease, this will appear from perusing the following illustrations." 173.

We are sorry we cannot afford space for the cases here alluded to.

*On the Morbid Changes of Typhus.*—Our author treats of these under the several heads of vascular system, glandular structure, membranous tissues, and parenchyma of organs. Under the first of these heads, he considers the state of the blood as well as of its vessels, and cites a number of facts and observations calculated, he thinks, to prove, that the constitutional symptoms arise from an impression on the vessels, and not merely from changes in the fluids.

"The almost certain order of the phenomena in typhus shews that the chief seats of the disease are involved by it in regular succession. There is, first, an affection of the head, then of the skin, afterwards of the lungs, and, lastly, of the bowels; which regular implication of different organs would lead to the inference that irritation rather travels through the vessels, than is excited by an impression from the blood, which would act nearly simultaneously on all parts of the vascular parietes." 186.

Dr. R. does not, however, deny that the blood is greatly altered in typhus.

"It is only by the supposition that some change of an injurious tendency takes place in the circulating fluids themselves, that we can offer an explanation of the symptoms by no means uncommon in certain cases during the prevalence of epidemic typhus. The cases to which this allusion refers, are those in which a fatal termination takes place about the third week, when the patients for a considerable time have been in a lethargic state, but are conscious when roused, and declare themselves free from pain. No one organ appears to suffer in an essential degree; the skin is perhaps hotter than natural, the pulse feeble, the tongue dry, hard, and black; the breathing may possibly be somewhat hurried, but auscultation can detect no disease of the lungs, nor is there any indication of essential mischief in the abdomen. Patients in this form of typhus will often take nourishment and occasionally exhibit signs of amendment. The fatal termination of these cases is very mortifying, as it is impossible to avoid entertaining expectation that they will terminate in recovery; there seems indeed no reason for an unsuccessful result; there are no symptoms of essential organic change to occasion it; no great excitement to exhaust the powers of life; still a serious constitutional disorder is excited, which our remedies are unable to remove, and which the efforts of nature are obviously quite inadequate to oppose: nor do examinations post-mortem, in these instances, throw any light upon the cause of death, &c. &c." 192.

We have already fully expressed our opinion of Dr. R.'s views respecting the part which vascular inflammation plays in the production of typhus. We pass on, therefore, to his observations on the pathology of the

*Glandular System.*—Under this head he classes the parotid gland, and the glands of the small intestines. The submaxillary gland also, the mesenteric, the axillary, and inguinal glands are all liable to be affected. Suppuration takes place sometimes in the substance of the parotid, sometimes in the cellular tissue around it. But the glandular follicles of the intestinal mucous membrane, are the parts chiefly affected. The lesions which they present, constitute, according to several eminent French pathologists, not merely the origin, but the very essence of "typhoid fever." But is the typhoid fever of France identical with the typhus of this country? Our author believes that it is; and yet, at the same time, he acknowledges, that "inflammation of the glands of the intestines is by no means a constant accompaniment of that form of typhus fever which is commonly met with in our country."

This is rather an unfortunate admission for one who argues, "that inflammation of the intestinal canal, when accompanied by ulceration, is one of the causes of phlebitis," and that phlebitis again is the cause of all the very grave symptoms of typhus.

The mucous membranes in typhus present, either separately or conjointly, various morbid alterations, the chief of which are staining, softening, inflammation, and ulceration. Under the head of mucous membranes, Dr. R. classes all those parts to which Meckel has extended the term, including the conjunctiva, *pia mater*, skin, &c.

Louis has observed that the mucous membrane of the epiglottis, glottis, larynx, and trachea is sometimes affected, though less frequently than other portions of the membrane lining the respiratory organs. Erysipelas, extending to the larynx and upper portion of the trachea, has more than once proved fatal in our author's practice. Bronchitis is of such frequent occurrence, that Hildenbrand considers it essential to the disease. Dr. Tweedie states that it is invariably present.

"The mucous membrane of the alimentary canal is very often disordered; not only does ulceration over the patches of glands destroy the tissue in this part, but in others also: thus the pharynx and œsophagus are occasionally ulcerated, a change which I have myself never met with, but which has been seen by M. Louis, who observes also that the stomach is in most cases more or less seriously implicated, being either softened, attenuated, changed in colour, or texture: still he declares that it was often natural, oftener indeed than in death from other causes." 211.

Of the genito-urinary portion of this membrane it may be remarked, that there is in some cases unnatural redness, and that symptoms of inflammation of the bladder occasionally show themselves.

Suffusion of the conjunctiva is a very constant phenomenon in typhus, and is especially interesting, if we may be allowed to infer that it indicates the state of circulation within the cranium. It appears early, simultaneously with the cerebral disturbance, or soon after.

But while the state of the conjunctiva thus indicates increased vascularity, the membranes of the brain furnish incontestable proof that, in very many cases, the vascularity there excited has gone on to positive inflammation. Out of 54 cases alluded to in the Cyclopædia of Medicine, 37 exhibited traces of inflammation of the brain. M. Louis admits that the *pia mater* was injected in half his cases. Our author believes that in every case there is increased vascularity of the *pia mater*, and that this probably is all that occurs in the more favorable instances, the vascularity subsiding without further mischief. In the more serious cases, however, when the patients have lived intemperately, or are of an irritable habit, the vascularity incident to the disease lights up into intense inflammation.

"All the characteristics of meningitis are met with in typhus; there is one impression, however, which I do not find mentioned in accounts of inflammation of the brain, several patients have told me that when labouring under this fever they have felt a strange sensation of doubt with respect to their own identity, imagining that their individuality was shared by others, and that they combined many beings in their single selves." 219.

The serous membranes are frequently implicated in typhus. Cerebral disturbance is often attributed to inflammation of the arachnoid, but our author is of opinion that the *pia mater* is the part generally affected. The following passage, relating to inflammation of the pleura, contains a highly important practical caution.

"The pleura is an important part, and with all the other tissues of the lungs is not unfrequently the seat of inflammation. It is a practical point of great moment to be aware of this fact, and to be impressed also with the knowledge that the inflammatory process goes on in this membrane without the usual admonition of the acute pain and hurried breathing which are so characteristic of ordinary pleurisy. Patients labouring under typhus will often remain in a state of apathy or indifference, and unconscious of all ordinary impressions; local disease therefore is wanting in outward demonstrations; thus pleurisy will occasion no pain, and there will be no cough, as the lungs will be sufficiently expanded by respiration to prevent congestion. I have known irreparable mischief produced before an idea was entertained that any disease existed in the chest, or that attention was drawn to the affected organ." 221.

But while we must be cautious not to overlook pleurisy, it is of still greater moment to be on our guard against the access of pneumonia.

"The comparative insensibility of the parenchyma of this organ (the lungs) will readily satisfy us of the absolute necessity of frequently resorting to auscultation, the use of which is signally exemplified in the treatment of typhus; for it is not only essential to ascertain the fact whether the lungs are inflamed, not only necessary to make out the character of the lesion, but of vital importance also to know the exact spot which is diseased, for there is no strength to be thrown away, and as our measures must be depleting, they must be appropriately directed. In my experience the substance of the lung is affected in almost all severe cases, scarcely has one patient passed through the protracted periods without oppression of the chest, hurried breathing, and small crepitation. The lungs, when examined after death, have presented the various stages of inflammation; and not only has consolidation been discovered at the base, to which part it is confined by M. Louis, but I have seen it also in the upper lobes. A purulent secretion is met with occasionally throughout the structure, and abscess will sometimes form in the upper, and indeed in every other part. In one case examined by me inflammation in the upper lobe of the right lung went on rapidly to ulceration, and large cavities were formed in this portion of the pulmonary tissue." 225.

Gangrene of the lungs is of very rare occurrence in typhus. This affection would deserve more consideration, if it were not for its extreme rarity.

'In the greater number of those who die from typhus, the substance of the brain is rosy and injected. This is not remarkable, considering the great vascularity of the *pia mater*, and the intimate connexion of this membrane with the brain, by means of its numerous minute vessels. Sir John Pringle states that he met with abscesses in the brain, and also in the cerebellum, and we learn from Hildenbrand that the brain will occasionally suppurate.

"Dr. Bateman considers that the inflammation in this fever is modified, except when it attacks the brain, but I do not see any reason to make an exception to the general rule, because, although the brain is often greatly affected, and though patients who have great disturbance in the functions of this organ, remain comatose for many days together, experience at times great difficulty of utterance, and suffer from effusion, as was exemplified in the case of Clarke, yet they will recover more completely from such affections when originating in typhus than is the case when similar results arise from idiopathic cerebral affection." 225.

Here terminates our author's description of the principal lesions which may take place during the progress of typhus. After some comments on the very numerous organic changes which its poison produces, and a few unimportant remarks on the classification of the disease, he then proceeds to consider the question of treatment, which he discusses at considerable length, and with the judgment of a man well versed in the difficulties of practice. His remarks on bloodletting, the use of opium, and of wine, are particularly worthy of the reader's notice. We regret, however, that he has not explained more fully the circumstances which warrant the employment of the last of these remedies, for there are few practical subjects deserving of more attention. We have known physicians who poured wine into their fever patients almost without measure—we need not say with what result; while others, withholding it entirely under circumstances favorable to its use, have also been very unfortunate in their practice, though not perhaps equally so.

We were among the number of those who hailed with pleasure the announcement of a work on typhus by Dr. Roupell. The physicians of our great metropolitan hospitals, with some honorable exceptions, have done comparatively little for the advancement of medical science. Their unrivalled opportunities for observation have too often been culpably neglected, and too often has the knowledge acquired from them been penuriously hoarded up, instead of being thrown, as it ought to be, into the general exchequer. These dignitaries of our profession, if we may so name them, have felt a proud reluctance to descend from their high stations, and compete in the race of science with men less fortunate, though perhaps equally deserving, as themselves. Many also, we fear, have been too busily occupied about the emoluments of practice, to devote much time to anything else. They have therefore rested satisfied with a local fame, when a more liberal spirit might perhaps have earned for them a wide-spread and lasting reputation. But a better feeling begins to prevail among them, and we can now number several of our metropolitan hospital physicians, among those who are most zealous and successful in the improvement and extension of medical science. Their names are not unknown to the profession, nor do they need either praise or mention from us. We have said that we were gratified when Dr. Roupell came forward to rank himself among the better and more liberal class of his compeers. On perusing his work, however, we could not help regretting that he had not withheld it from the public until he had corrected those faults, and removed those imperfections, of which, in the preface, he expresses himself to be fully sensible. A longer contemplation of his subject, and the exercise of a little more judgment, might have enabled him to condense his "short treatise" of 301 pages, into one hundred, or one hundred and fifty pages at most, with eminent advantage, at the same time, as regards both clearness and accuracy. Many long and impertinent quotations, which now serve only to parade learning, would, no doubt, have been excluded, if our author had bestowed more time and consideration on his work. Mature reflection too must have taught him that to build vain theories upon the frail foundations which imagination furnishes, is an occupation better adapted to pupils, and inexperienced candidates for practice, than to sober teachers, and physicians long and intimately acquainted with disease. Indeed it is high time altogether to banish from medicine the pernicious practice of theorizing from assumed data. In no other science, we believe, would such a practice be tolerated. Why, then, should it be suffered to continue in medicine?

If we have spoken our opinion freely of Dr. R.'s work, and in many places ventured to dissent both from his doctrines and his statements, we have been influenced solely by a sense of our duty as public journalists; and we have in every instance endeavoured to avoid all possible misrepresentation by laying his own words before our readers, who are therefore fully enabled to judge, whether, in any instance, we have censured without sufficient reason.

But notwithstanding our just dissatisfaction with Dr. Roupell's present treatise, we doubt not in the least his capacity to write a good work on typhus. Let him add to his practical knowledge of the disease, a more cautious and philosophical spirit in examining, selecting, and estimating facts; let him pay more attention to arrangement, to brevity, and accuracy of expression, and we have no doubt that he may re-write and remodel his work, so as to make it really worthy the notice of his professional brethren.

